# Lack of concordance among infant social attention measures

**DOI:** 10.1038/s41598-026-36807-5

**Published:** 2026-01-20

**Authors:** Charlotte Viktorsson, Kim Astor

**Affiliations:** 1 Development and Neurodiversity Lab, Department of Psychology, Uppsala University, Uppsala, Sweden; 2 Uppsala Child and Baby Lab, Department of Psychology, Uppsala University, Uppsala, Sweden

**Keywords:** Social attention, Gaze following, Eye looking, Face preference, Infants, Human behaviour, Social behaviour, Social neuroscience

## Abstract

Infants’ tendency to focus on socially relevant cues is commonly referred to as ‘social attention’ and is often conceptualized as a unified construct. However, few studies have examined the concordance among measures that fall under this label. In this study, we assessed 50 ten-month-old infants using three common measures of social visual attention: eye (versus mouth) preference, gaze following, and face preference (versus non-social objects). We also examined their association with concurrent parent-rated socio-communicative abilities. No significant associations were found among the gaze measures. Notably, only eye preference was uniquely associated with communicative abilities (β = 0.364, *p* = 0.018), while the other gaze measure showed no such relationship. These findings suggest that what is commonly referred to as ‘social attention’ reflects a set of functionally distinct and seemingly unrelated behaviors, highlighting the need to re-evaluate its conceptualization as a unified construct in early development.

**Supplementary Information:**

The online version contains supplementary material available at 10.1038/s41598-026-36807-5.

## Introduction

Selective visual attention represents one of the earliest mechanisms through which infants engage with and learn from their environment, and from a young age, children show a marked preference for social stimuli (e.g.^1–3^). Already at birth, newborns preferentially orient towards faces^4^, and this preference for faces increases continuously throughout the first year of life^5^. Individual differences in infants’ selective attention to faces are predictive of later outcomes. For example, a stronger preference for images of static faces over non-social objects at five months has been associated with later language comprehension^6^.

When looking at dynamic faces, infants generally prefer looking at the eyes of another person in the first six months of life (e.g.^7^), a preference that shifts toward the mouth in the latter half of the year (e.g.^8^). In line with this preferential attention, a tendency to look at the eyes at five months has been found to predict later language comprehension^7^, while increased attention to the mouth in the second half of the first year has been associated with concurrent and later social and communicative skills^9–11^.

Another milestone in social development is the ability to synchronize visual attention with others towards external objects, enabling infants to direct focus towards the most relevant information for their learning and environment. This ability, known as gaze following, is central to attention sharing and social cognition^12,13^ and has been linked to both language development (see^14^ for a systematic review) and later socialization behaviors^15^.

While face preference, early eye-vs-mouth looking, and the emergence of gaze following each follow distinct developmental trajectories, they share a common functional aim: to guide infants’ visual attention toward socially meaningful information. This tendency to focus on socially relevant cues is commonly referred to as ‘social attention’ and is often conceptualized as a single, unified construct (e.g.^16^). Framing these patterns of looking as facets of a broader social attentional system help explain their collective role in scaffolding later social and communicative development. In this sense, social attention in infancy is often regarded as early social competency.

Consistent with the view of social attention as a unified construct are studies demonstrating that visual attention to the mouth of a talking person and the tendency to follow gaze is moderately correlated (*r* = 0.26) in 12-month-olds^10^, suggesting that these behaviors may stem from a shared drive for social engagement. At the same time, individual differences indicate that this system is not monolithic as children vary widely in how they attend to social stimuli^6,7,17^. A recent twin study of more than 500 five-month-olds showed that both eye preference (versus looking at the mouth) and face preference (versus looking at non-social objects) are largely heritable, yet only weakly correlated (*r* = 0.11), and appear to be shaped by distinct genetic factors^6^. This challenges the view that all social looking behaviors stem from a single underlying trait and instead suggests they emerge from partially independent underlying mechanisms and are shaped by separate evolutionary pressures^18^.

Traits that are initially unrelated may become correlated over time^19^, which might explain the lack of association among social attention measures at 5 months^6^ and the moderate association found later at 12 months^10^. This view aligns with neurodevelopmental accounts suggesting that the social brain is not yet matured in infancy^20,21^. Although regions such as the STS, fusiform gyrus, and amygdala are active in infants during processing of social information, these responses are not yet fully integrated and socially specialized. At the same time, research on the concordance among measures frequently labeled as ‘social attention’ is scarce, highlighting the need for a more detailed investigation of this foundational construct. In this study, we aimed to examine the association between three commonly used eye-tracking measures of social attention: the eye-mouth-index (EMI; i.e., eye versus mouth preference), gaze following (GF), and face preference (versus non-social objects) in 10-month-old infants, an age at which they reliably exhibit a preference for faces^5^, demonstrate the ability to follow gaze^22^, show robust differentiation in attention to the eyes versus the mouth^8^, and are thought to possess a more advanced understanding of others as intentional social beings^23,24^. We hypothesized that we would find a statistically significant correlation among all three measures of social attention. We expected to find a positive association between gaze following and face preference and a negative association between EMI and gaze following (meaning that more gaze following is associated with more mouth-looking, in line with earlier findings in 12-month-olds^10^. However, we had no directional hypothesis regarding the association between EMI and face preference. Without correlations among these three measures, it is difficult to regard them as reflecting a unified construct of social attention in the way they are typically portrayed in the literature: as indicators of an early social competency.

A unified dimension of social attention would suggest that these measures are similarly associated with social behaviors. Therefore, we also examined whether these measures relate to concurrently measured parent-rated socio-communicative abilities. We hypothesized that we would find a significant association between all three measures of social attention and socio-communicative abilities. We expected the associations between socio-communicative abilities and gaze following/face preference to be positive, but we had no directional hypothesis regarding the association with EMI. All hypotheses were pre-registered at OSF (https://osf.io/dsvkf/).

## Methods

### Participants

Participants were recruited at the Child and Babylab at Uppsala University, from a list of families who has previously expressed interest in participating in research with their child. The families were contacted via email or by phone, and then invited to the lab for the eye tracking session. Infants were included only if their age was 10 months (+/- 2 weeks), they had no uncorrected visual or hearing impairment, they were born at week 37 or later, and they had no traumatic brain injury or neurological condition. After the eye tracking session, which lasted for approximately 8 min, the caregiver filled in questionnaires on demographic information and child behavior. In total, 53 infants participated in the study. Three infants were subsequently excluded due to technical issues with the eye tracker, and the final sample (with valid data in at least one of the conditions) was 50 children. Demographic information is provided in Table 1. The study was approved by the Swedish Ethical Review Authority and was conducted in accordance with the Declaration of Helsinki. Written informed consent was obtained from all caregivers.

**Table 1:** Demographic information (*N* = 50).

	Mean (SD)^a^ [Min; Max]
N females (%)	28 (56.0%)
Age (in days) at assessment	301.58 (8.48) [288; 320]
Family income^b^	6.68 (2.04) [2; 10]
Maternal education level^c^	4.72 (0.76) [3; 6]
Paternal education level^c^	4.56 (1.07) [1; 6]
Number of children in the household	1.60 (0.78) [1; 4]

^a^ Except for N females, which shows the frequency.

^b^ Family income per month. Scale 1–10 where 1 = < 20 K, 2 = 20–29 K, 3 = 30–39 K, 4 = 40–49 K, 5 = 50–59 K, 6 = 60–69 K, 7 = 70–79 K, 8 = 80–89 K, 9 = 90–99 K and 10 = > 100 K (SEK).

^c^ Education level, on a scale from 1 to 6, where 1 = Primary, 2 = 2-year Secondary, 3 = 3-4-year Seconday, 4 = Up to 2 years of University/Higher vocational education, 5 = Bachelor’s degree/Master’s degree/Higher vocational education, and 6 = Licentiate/doctoral degree.

### Eye tracking measures

Gaze data was recorded using a Tobii TX300 eye tracker (120 Hz) and a Tobii Pro Spectrum (120 Hz), each with a standard screen (24”), 1920 × 1080 pixels. Of the total sample, 41 were tested using the TX300 eye tracker, and 9 were tested using the Tobii Pro Spectrum eye tracker (due to sudden technical issues with the TX300 towards the end of the data collection). The infant was seated in their parent’s lap, approximately 60 cm from the screen. Before the eye-tracking session, a 5-point calibration session was conducted.

Before analyzing the gaze data, each participant’s data at all instances of attention-grabbers (salient stimuli presented in the middle of the screen between trials) were evaluated via ocular inspection, and a simple linear transformation of data was performed when linear drifts were detected (using custom MATLAB scripts).

#### EMI condition

**Fig. 1:**
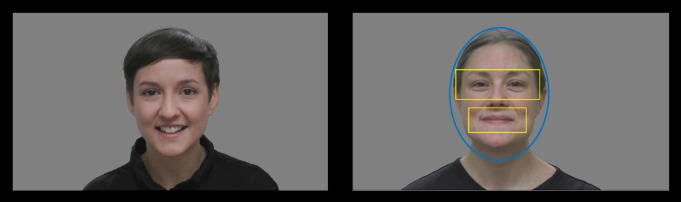
Still frames from two of the videos included in the EMI condition, depicting the two actors. The AOIs for face, eyes, and mouth are plotted to the right. Both actors have provided written informed consent to publish identifying images of them.

In this condition, infants were presented with 12 videos of two different women singing common Swedish nursery rhymes (each woman appeared in six videos). These videos have been previously used in studies of infant gaze patterns (e.g.^7^). AOIs were created by first analyzing the videos using the open-source software OpenFace, which detects faces via computer vision and extracts facial landmarks^25^. We then used the x- and y-coordinates in pixels for different facial landmarks to create dynamic AOIs for each frame of the videos. The eye AOI was 510 × 180 pixels, and the mouth AOI was 350 × 150 pixels (see Fig. 1). The horizontal radius of the face AOI was 300 pixels and the vertical radius of the face AOI was 400 pixels. The EMI was calculated based on total looking time at the eyes divided by total looking time at both eyes and mouth (scale 0–1, where 0 means looking exclusively at the mouth and 1 means looking exclusively at the eyes). Trials were excluded if the infant looked at the screen for less than 2.5 s, and at least four valid trials were needed in order to be included in the EMI analyses.

#### GF condition

**Fig. 2:**
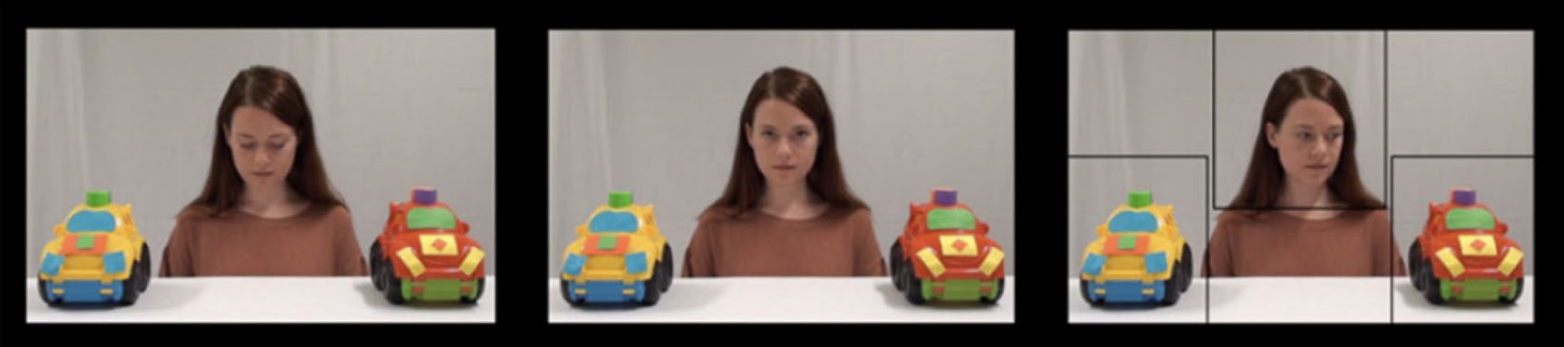
Still frames from one of the videos in the GF condition. The AOIs are shown to the right. This figure is republished from Astor et al. (2020) with permission from authors. When the stimuli was originally created, the actor provided consent to publish identifying pictures of her.

In this condition, the infants viewed 6 videos depicting a woman looking at the camera before looking at one of the objects in front of her. A ‘beep’ sound was played before the woman looked at the camera. Three rectangular AOIs were created: one covering the actor’s face and two covering the toys (see Fig. 2). GF was assessed using the first look paradigm, where the infant’s initial gaze shift from the actor to either toy was recorded as either congruent or incongruent. GF was calculated by subtracting the number of incongruent trials from the number of congruent trials, resulting in a difference score. Over six trials, this score ranged from − 6 to 6, with a positive score indicating greater GF. A trial was classified as valid if the child looked at the face and then at either of the objects. At least two valid trials were needed in order to be included in further analyses.

#### Face preference condition

**Fig. 3:**
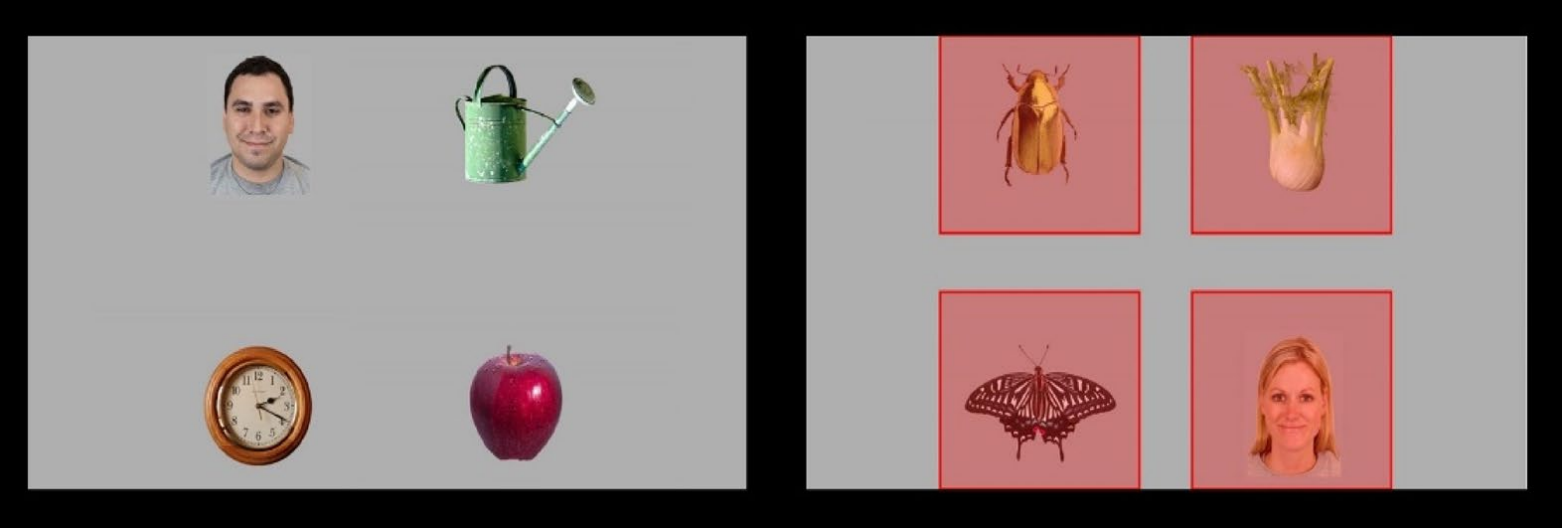
Two of the complex displays shown in the face preference condition. The AOIs are shown to the right. Written permission to publish this figure was obtained from the Chicago Face Database.

In this condition, infants were shown 8 arrays of complex displays (see Fig. 3), each shown for 8 s. These arrays included one face (with direct gaze, and with its location in the array counterbalanced between trials), and three non-face objects. Half of the arrays included a female face and half of them included a male face, every array included a unique face not present in any other stimuli. These images were created using the Chicago Face Database^26^. The non-face objects consisted of both objects that are familiar to most children (e.g., a ball, a clock, and a lamp) and objects that may be unfamiliar at this age (e.g., a beetle, a crystal, and an umbrella). Before each array, an attention grabber was presented in the middle of the screen. The AOIs were 530 × 470 pixels, and covered all objects in the array (Fig. 3). Face preference was calculated as the total looking time at the face divided by the total looking time at the face and all objects (scale 0–1, where 0 means no looking time at the face and 1 means that the child looked exclusively at the face). Trials were excluded if participants looked at the screen for less than 3 s. At least two valid trials were needed in order to be included in further analyses of the face preference condition.

### Parent-rated measure

Socio-communicative abilities were measured using the Infant-Toddler Checklist (ITC;^27^), which is a 24-item parent-rated questionnaire, used to identify children with any type of socio-communicative delay. Higher scores indicate a higher degree of socio-communicative abilities. Items include, for example, questions on whether the parent knows when the child is happy or sad, and whether the child lets the parent know when they need help reaching an object. We used the total score as the primary measure of socio-communicative abilities. We also extracted composite scores on the communication, expressive speech, and symbolic subscales.

### Statistical analyses

We examined the associations between each looking measure by calculating Pearson’s r. As per the analysis plan, a confirmatory factor analysis (CFA) and a calculation of Cronbach’s alpha for the three measures were planned. However, this would only be performed if significant correlations were found between the measures.

In addition, we examined the associations between each looking measure and concurrent socio-communicative abilities by calculating Pearson’s r for each association.

The EMI was not associated with sex (*r* = -0.007, *p* = 0.965), age (*r* = -0.007, *p* = 0.960), family income (*r* = -0.003, *p* = 0.984), maternal education level (*r* = -0.266, *p* = 0.071), paternal education level (*r* = 0.053, *p* = 0.722), or number of children in the household (*r* = 0.134, *p* = 0.370). The difference score in the gaze following condition was not associated with sex (*r* = 0.220, *p* = 0.157), age (*r* = -0.039, *p* = 0.806), family income (*r* = 0.089, *p* = 0.571), maternal education level (*r* = 0.084, *p* = 0.591), paternal education level (*r* = -0.006, *p* = 0.971) or number of children in the household (*r* = -0.089, *p* = 0.568). Similarly, face preference was not associated with sex (*r* = -0.119, *p* = 0.410), age (*r* = -0.275, *p* = 0.053), family income (*r* = 0.142, *p* = 0.324), maternal education level (*r* = 0.200, *p* = 0.163), paternal education level (*r* = 0.163, *p* = 0.259), or number of children in the household (*r* = -0.173, *p* = 0.229). Based on these results, no covariates were included in further analyses.

## Results

Descriptive statistics on eye tracking measures and the ITC are presented in Table 2 (see Supplementary Information S1 for distributional plots of the primary gaze measures and Supplementary Information S2 for distributional plots of the ITC). The number of included trials for each condition was not associated with the EMI (*r* = -0.186, *p* = 0.344), GF (*r* = 0.208, *p* = 0.181), or face preference (*r* = 0.040, *p* = 0.783).

The mean EMI (0.62) was significantly different from 0.50 (t(46) = 3.267, *p* < 0.001, Cohen’s d = 0.477), meaning that, at a group level, the infants looked preferentially more at the eyes than at the mouth. The mean GF difference score (1.81) was significantly different from zero (t(42) = 5.534, *p* < 0.001, Cohen’s d = 0.844), meaning that, at a group level, the infants displayed a tendency to follow the gaze of the actor. The mean percentage of looking time at the face in the face preference condition was 0.53, which was significantly different from 0.25 (i.e., chance level; t(49) = 14.580, *p* < 0.001, Cohen’s d = 2.062), indicating a preference for looking at the face over the non-social objects.

**Table 2:** Descriptive statistics on eye tracking measures and the ITC.

	Mean (SD) [Min; Max]
EMI	
Looking time at screen (seconds)	8.29 (1.73) [3.48; 10.17]
Looking time at face (ratio)	0.93 (0.10) [0.50; 0.99]
Eye-mouth-index	0.63 (0.26) [0.11; 0.99]
N valid trials	11.45 (1.44) [5; 12]
GF	
GF difference score	1.81 (2.14) [-4; 6]
N valid trials	5.19 (1.19) [2; 4]
Face preference	
Looking time at screen (seconds)	6.75 (1.20) [3.45; 8.07]
Looking time at face (seconds)	3.22 (1.15) [1.18; 5.95]
Looking time at face (ratio)	0.53 (0.14) [0.21; 0.85]
N valid trials	7.50 (1.22) [3; 8]
ITC	
Total score	27.14 (6.37) [14; 49]
Communication subscale	14.68 (3.89) [7; 24]
Expressive speech subscale	6.06 (1.80) [2; 12]
Symbolic subscale	6.40 (2.08) [1; 13]

EMI = eye-mouth-index, GF = gaze following, ITC = Infant-toddler checklist.

There was no significant association between the EMI and GF (*r* = -0.196, *p* = 0.207), between EMI and face preference (*r* = -0.103, *p* = 0.489), or between GF and face preference (0.036, *p* = 0.818). Due to the lack of correlations across measures, we did not move on to a factor analysis or a Cronbach’s alpha. Bayesian analyses suggested moderate support for the null hypothesis with regards to all three correlations (EMI vs. GF: BF_01_ = 3.809, EMI vs. face preference: BF_01_ = 6.915, GF vs. face preference: BF_01_ = 8.183).

There was no significant association between the EMI and ITC (*r* = 0.276, *p* = 0.061), GF and ITC (*r* = 0.098, *p* = 0.533), or face preference and ITC (*r* = -0.244, *p* = 0.087). In light of these findings, we added a number of analyses to further examine potential associations between early gaze behaviors and socio-communicative abilities. None of the following analyses were pre-registered, starting with a linear regression model including all gaze measures as predictors and ITC total score as outcome variable. The regression model was significant (F(3,39) = 2.924, *p* = 0.046, R^2^ = 0.184), and the EMI was a significant positive predictor of ITC (β = 0.357, *p* = 0.022). Neither GF (β = 0.174, *p* = 0.245) nor face preference (β = -0.180, *p* = 0.228) were significant predictors of ITC. To further examine the association to socio-communicative abilities, we added all gaze measures to three linear regression models, with the three subscales of ITC as outcome variables. The regression model with the communication subscale as outcome was significant (F(3,39) = 3.370, *p* = 0.028, R^2^ = 0.206). The EMI was a significant predictor (β = 0.364, *p* = 0.018), but neither GF (β = 0.252, *p* = 0.092) nor face preference (β = -0.171, *p* = 0.245) were significant predictors. The regression model with expressive speech as outcome variable was not significant (F(3,39) = 0.787, *p* = 0.509) and neither was the regression model with the symbolic subscale as outcome (F(3,39) = 1.902, *p* = 0.145).

### Additional analyses

As prompted by a reviewer, we additionally calculated the number of times each infant first fixated on the face in the face preference task. Fixations were extracted using the fixation detection algorithm I2MC^28^. Fixation durations were only considered valid if the temporal duration was 100 milliseconds or longer. We then calculated the ratio of first looks to the face (relative to the number of valid trials for each participant). The mean ratio was 0.512 (SD = 0.228, Min = 0.125, Max = 1.00). There was no statistically significant association between EMI and ratio of first looks to faces (*r* = -0.034, *p* = 0.822) or between gaze following and ratio of first looks to faces (*r* = -0.072, *p* = 0.646).

## Discussion

In this study of 10-month-old infants, we examined whether three standard measures of visual social attention reflect a common dimension and whether they are related to concurrent socio-communicative behaviors as rated by their caregiver. We found no associations across the tendency to look at eyes versus mouth, the tendency to follow gaze, and the preference for looking at faces (versus non-social objects). Bayesian analyses suggested moderate support for the null hypothesis across these correlations. These findings, together with behavioral genetic evidence, support a broader theoretical reconsideration of social attention in infancy. Specifically, our results call for a more nuanced understanding of the behaviors that fall under this umbrella term. Rather than reflecting a unified construct (e.g.^16^), social attention may be better understood as an umbrella term for multiple functionally distinct behaviors with separate developmental trajectories (e.g.^18^). This interpretation aligns with recent behavioral genetic findings showing that different genetic factors influence eye and face preferences in early infancy^6^, indicating that different social attention measures reflect different underlying processes.

While these behaviors may draw on innate sensitivities to social agents as proposed by core knowledge theories^29^, the lack of coherence at 10 months suggests that these sensitivities are expressed through diverse pathways rather than a unified system. Future work should explore how and when these behaviors begin to align and whether such alignment is driven by neural maturation, social learning, or interactive feedback from communicative success. From a neurodevelopmental perspective, the social brain is still maturing at 10 months and is not yet functionally integrated^20,21^. Within this framework, early activations in regions such as the STS, fusiform gyrus, and amygdala reflect broadly tuned, perceptual responses to social stimuli that serve as precursors of later social specialization. Functional connectivity among these regions increases gradually across the first years of life, supporting a transition from domain-general perceptual responsiveness to selective, socially specific processing^21^. In other words, early activation in these regions does not necessarily imply shared representational meaning.

It is further possible that a lack of association among these gaze behaviors could be due to low-level domain-general visual processing styles, which affect these measures differently. For example, a recent study in adults found that the EMI is highly correlated with vertical looking preference on non-social stimuli^30^, suggesting that an eye or mouth preference merely reflect a preference for upper or lower stimuli, regardless of social saliency. Whether this pattern extends to infants remains to be tested. Gaze following, in turn, might be affected by sensitivity to certain perceptual cues, such as the spatial distance between the actor and the object^31–33^, or motion towards an object^22,34,35^. Domain-general processing across these measures does not imply that they should correlate, because the relevant perceptual or attentional processes may not rely on the same underlying mechanisms. If both domain-general and domain-specific mechanisms jointly govern these behaviors, then accounting for these perceptual biases or processing styles may reveal associations that are otherwise obscured. In other words, once low-level attentional tendencies are measured and controlled for, the relationship among social attention measures could strengthen and more clearly reflect socially driven attention. Identifying and modeling these domain-general influences therefore represents an important direction for future research.

While Tenenbaum et al.^10^ found a moderate association between mouth preference and gaze following at 12 months of age, it is important to note that both measures in their study were derived from responses to the same set of stimuli videos. The gaze measures in the present study were derived from separate sets of stimuli, each of which has either been repeatedly used in previous studies (EMI and gaze following) or is closely modeled on well-established stimuli (face preference) that have reliably produced robust effects^6,7,36^. While we did not find any significant associations across these measures at 10 months of age, it remains possible that different social attention behaviors become correlated over time^19^. Neurodevelopmental accounts emphasizing immature integration of the social brain network predict weak or absent correlations early in life, with stronger coherence emerging as connectivity strengthens. A key limitation is that the present study includes infants at only one age. Consequently, our results cannot determine whether the lack of concordance at 10 months reflects a persistent fractionation of social attention or whether stronger associations emerge later in development. Distinguishing between these alternatives requires either longitudinal designs or cross-sectional samples spanning a broader age range.

The developmental dissociation among gaze measures in our study was further reflected in how they related to concurrent socio-communicative abilities more broadly. We examined behaviors such as whether the infant tries to get their caregiver’s attention, whether they wave to greet people, and whether they try to make the caregiver laugh, as captured by the ITC scale. After controlling for gaze following and face preference, we found that only the eye-mouth-index (EMI) was uniquely related to concurrent communicative behaviors with a positive association between preference for eyes and concurrent communicative behaviors. Although gaze following has previously been linked to later language outcomes in toddlerhood(^14^, though see^37^ for a critical appraisal), the ITC items at this age reflect many behaviors related to initiating joint attention. This ability is, perhaps somewhat surprisingly, not associated with the ability of responding to joint attention, which gaze following is an example of^38^. The tendency to look at eyes versus the mouth may instead be more closely linked to the mechanisms underlying initiating joint attention, which could explain the lack of association between the EMI and gaze following. However, this interpretation remains speculative and warrants further investigation.

An alternative explanation to the lack of concordance among the social attention measures is that they reflect different expressions of social preferences rather than social competences. For instance, infants might prefer looking at the eyes or the mouth, both socially relevant stimuli, even if they differ in informativeness. While this argument may hold some merit for the EMI, it is more difficult to apply to the other measures: Infants do disengage from faces, and when they do, some synchronize visual attention through gaze following while others do not. At this point, there are no competing socially relevant targets comparable to the eye–mouth distinction, and thus no alternative “social” way of looking, only visual synchronization. The same reasoning applies to face preference, where no other social stimulus is present. Therefore, while the EMI could arguably reflect individual differences in social preference, this reasoning cannot explain the lack of association between face preference and gaze following. Moreover, the literature treats these social attention measures as indicators of early social competence. Under this conceptualization, they should reflect a shared underlying construct, and their lack of correlation is therefore theoretically problematic. Even if one interprets the three measures as tapping partially distinct facets of social processing, at least moderate correlations should be detectable under the assumption that they are indicators of a unified dimension implying social competence. Bayesian analyses instead provided evidence in favor of the null hypothesis for all pairwise associations, supporting the conclusion that these measures do not operate as a single unified construct at 10 months. However, it remains possible that the measures reflect genuinely distinct facets of social attention. These facets may vary in the extent to which they depend on domain-general versus domain-specific processes: some behaviors may be shaped by combinations of preference, perceptual biases, and processing speed, whereas others may more directly reflect social understanding or social motivation. Under this view, a specific social preference need not map onto social competence, and dissociation across measures would not necessarily imply a failure of any single construct but rather the presence of multiple, distinct components of early social attention. Consequently, it may be more appropriate to discuss these measures in terms of the specific processes they index, rather than treating them broadly as social attention.

The lack of concordance among these measures may also have implications for research on neurodevelopmental conditions, such as autism. If different gaze measures do not cohere even in typical development, it will be important for future work to examine whether atypical performance on one measure predicts difficulties on others, or whether different measures might identify distinct autistic subgroups with differing developmental pathways. Such work could also clarify whether interventions should target specific components of social attention rather than assuming a single underlying mechanism, and whether trajectories of concordance across measures differ between autistic and neurotypical children.

While we included three frequently used gaze measures of infant social attention, our study is not covering all measures that have been labelled as ‘social attention’. For example, earlier eye tracking studies have also focused on how infants view interaction among other children^39^, how they view complex social scenes^40^, and how they respond to bids for joint attention in a live situation^41^. A more comprehensive analysis of early social looking behaviors might shed light on whether there are other measures of social attention that may be intercorrelated. However, it is worth noting that the measures included in this study are well-known and widely used in the field of infant eye tracking.

A limitation of the current study is the relatively small sample size. While we would expect moderate-to-large associations among measures belonging to unified construct, we did not have power to detect potential small-to-moderate effects. We included Bayesian analyses suggesting moderate support for the null hypothesis, but future studies should recruit a larger and more diverse sample, in order to examine whether there might be small (but significant) associations across these social attention measures.

## Conclusions

How infants orient to and scan faces, and how they follow the gaze of other people, are behaviors often referred to as ‘social attention’, though attempts to assess them as a unified construct remain scarce in the literature. In this study, we found no statistically significant associations between any of these measures. Furthermore, only the tendency to look at eyes (versus mouth) was uniquely related to concurrent communicative abilities, while no association to other gaze measures was found. These findings suggest that the concept of ‘social attention’, as currently defined, reflect fragmented behaviors, and a growing body of research suggests that much of what has been labeled as social attention may instead be driven by domain-general perceptual or attentional mechanisms. Together, these findings call for a re-evaluation of the notion that social attention represents a unified dimension early in development.

## Supplementary Information

Below is the link to the electronic supplementary material.

Supplementary Material 1

## Data Availability

The analyses presented here were preregistered at OSF (https://osf.io/dsvkf/). The data and code necessary to reproduce the analyses presented here are not publicly accessible, but will be made available upon reasonable request to the corresponding author. Note that sharing of pseudonymized personal data will require a data sharing agreement, according to Swedish and EU law.
