# Supplementary material for: Lack of concordance among infant social attention measures
Source: Sci Rep. 2026 Jan 20;16:2591. doi: 10.1038/s41598-026-36807-5 (PMC12819393; doi:10.1038/s41598-026-36807-5)
Supplement: Supplementary file 1 — Supplementary Material 1 [file 41598_2026_36807_MOESM1_ESM.docx]

Supplementary Information

**Lack of Concordance Among Infant Social Attention Measures**

Charlotte Viktorsson^1*^ & Kim Astor^2^

^1^Development and Neurodiversity Lab, Department of Psychology, Uppsala University; Uppsala, Sweden

^2^Uppsala Child and Baby Lab, Department of Psychology, Uppsala University, Uppsala, Sweden

*Corresponding author. Email: charlotte.viktorsson@psyk.uu.se

**Supplementary Information S1**. Distributional plots of gaze measures.


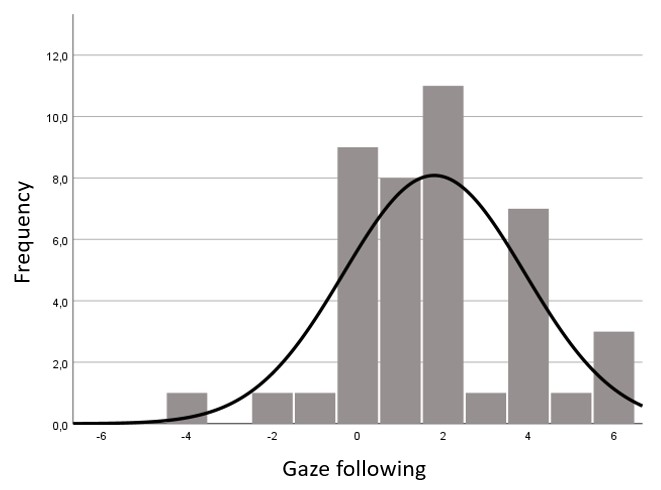


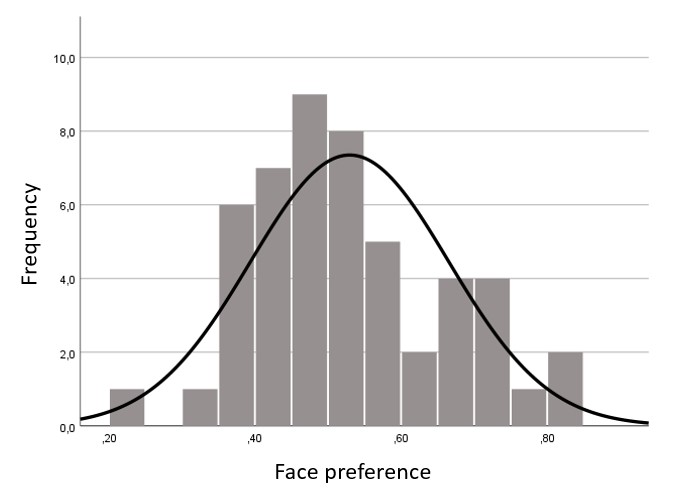


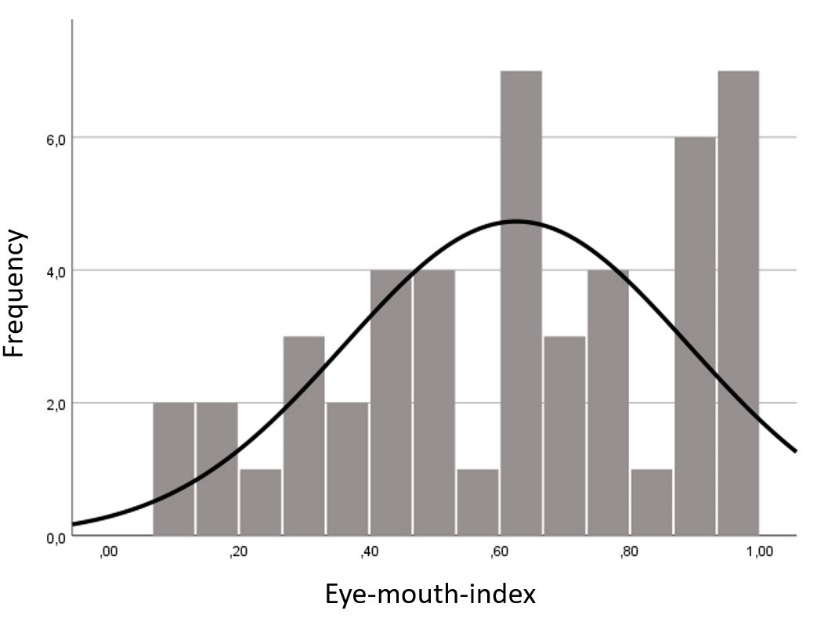


**
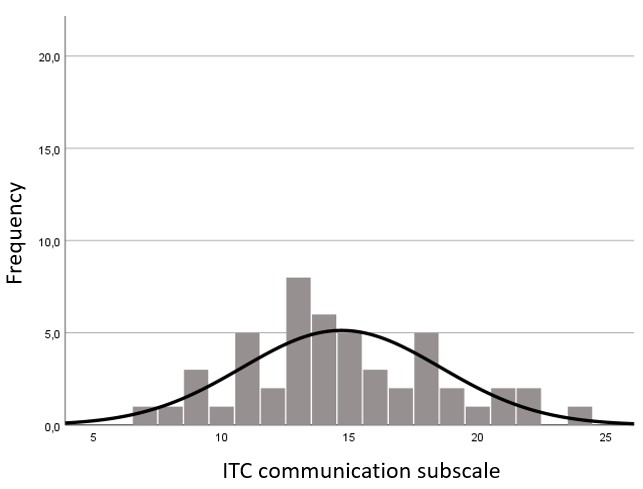

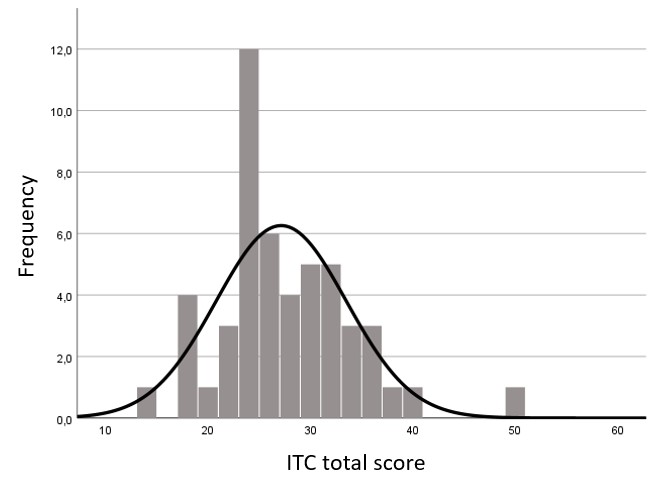

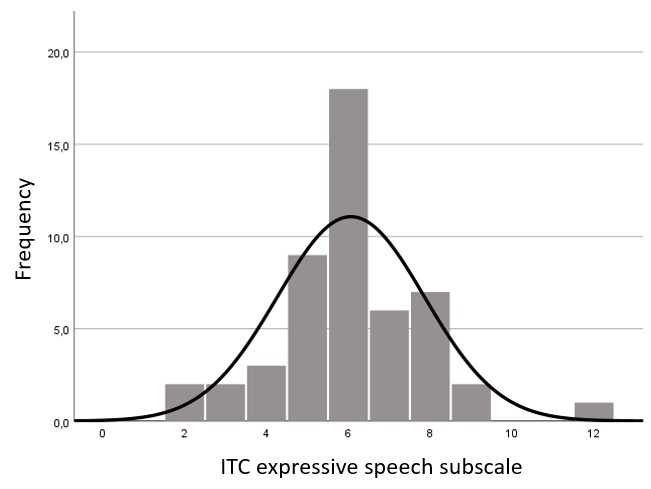

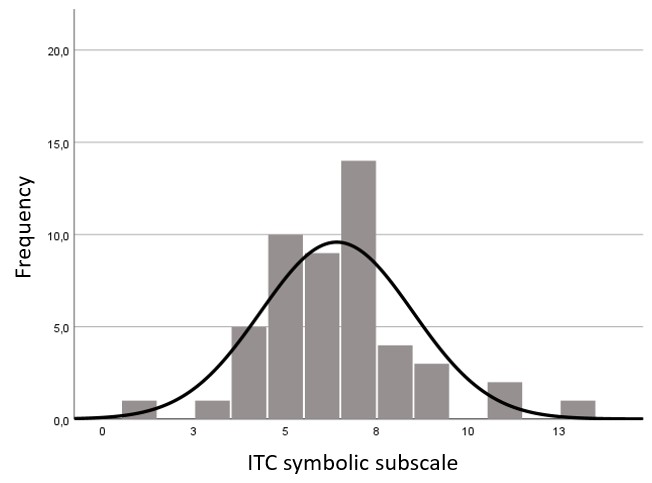
Supplementary Information S2**. Distributional plots of the ITC.
